# Relationship between carotid atherosclerosis and lipoprotein (a) in patients with acute ischemic stroke

**DOI:** 10.3389/fneur.2024.1383771

**Published:** 2024-06-26

**Authors:** Yongna Zhao, Zhichao Wang, Ruijun Ji, Yongjun Wang, Yaguang Zhang, Kai Yu

**Affiliations:** ^1^Department of Neurology, Renqiu Kangji Hospital, Renqiu, China; ^2^Department of Neurology, Beijing Tiantan Hospital Affiliated to Capital Medical University, Beijing, China

**Keywords:** acute ischemic stroke, lipoprotein (a), blood lipid, carotid artery atherosclerosis, cholesterol

## Abstract

**Objective:**

This study aimed to examine the relationship between lipoprotein (a) (Lp[a]) and other blood lipid indexes and carotid artery atherosclerosis in patients with acute ischemic stroke (AIS).

**Methods:**

A total of 2,018 patients were selected from the hospital “acute stroke intervention and secondary prevention registration database” by identifying blood fat indexes (cholesterol, triglyceride, high-density lipoprotein cholesterol, low-density lipoprotein cholesterol and Lp[a]). Based on the results of carotid artery ultrasound examinations, the patients were divided into a “no plaque” group, comprising 400 patients, a “plaque and no stenosis” group, comprising 1,122 patients and a “carotid stenosis” group, comprising 496 patients. The relationship between Lp(a) and blood lipid indexes and carotid artery atherosclerosis was then investigated using multi-factor logistics regression analysis.

**Results:**

There were 400 patients (19.8%) with no carotid plaque, 1,122 patients (55.6%) with plaque and no carotid stenosis and 496 patients (24.6%) with carotid stenosis. As the degree of carotid artery atherosclerosis increased, the Lp(a) level gradually increased; Lp(a) and cholesterol were identified as independent risk factors for carotid atherosclerosis.

**Conclusion:**

Lipoprotein (a) and cholesterol are independent risk factors for patients with AIS with carotid atherosclerosis, and their levels increase with the degree of carotid artery atherosclerosis; therefore, attention should focus on levels of cholesterol and Lp(a) in acute stroke patients to control atherosclerosis effectively.

## 1 Introduction

Acute ischemic stroke (AIS) accounts for 60–80% of all stroke cases in China (1), and the recurrence of AIS is high, with an annual rate of 17.7% (2). Secondary prevention should be initiated as soon as possible after the acute onset of this disease. This prevention strategy targets both modifiable and nonmodifiable stroke risk factors, and the interaction of these risk factors contributes to the development of arteriosclerosis, which in turn induces stroke. Lipid metabolism disorder, especially the metabolism of cholesterol and low-density lipoprotein (LDL), is one of several established modifiable stroke risk factors, and intensive statin therapy is recommended to reduce the levels of LDL and cholesterol to achieve the goal of secondary prevention. Targeting modifiable risk factors, such as lipid disorders (especially cholesterol and LDL), has been central to reducing stroke incidences. Intensive statin therapy has been widely endorsed in this regard. Recent studies have also identified lipoprotein (a) (Lp[a]) as a critical cardiovascular risk factor, extending beyond traditional lipid measures to include genetic predispositions that enhance cardiovascular risks (3).

Lipoprotein (a), a glycoprotein synthesised in the liver, consists of LDL-like particles and apolipoprotein A (Apo[a]) (4). Studies and meta-analyses on cardiovascular diseases have shown that Lp(a) is an independent risk factor for cardiovascular diseases (5) and also an independent predictor of coronary heart disease (CHD) (6). Studies have found that elevated serum Lp(a) is associated with an increased risk of cardiovascular and cerebrovascular diseases (7), and high serum Lp(a) is an independent risk factor for cardiovascular and cerebrovascular diseases, atherosclerotic obliterans and deep vein thrombosis (8). However, no consistent conclusion has been reached (9), and the findings of studies on Lp(a) and cerebrovascular diseases are inconsistent (10). In addition, only a high level of Lp(a) is detected in blood lipid testing in a large number of patients with acute and recurrent strokes in clinical practice. Despite its established role in cardiovascular risk, the implications of Lp(a) in carotid atherosclerosis among patients with AIS remain underexplored, presenting a gap that this study aims to fill. This is particularly pertinent as Lp(a) has been associated independently with increased risks of atherosclerosis and subsequent cardiovascular events, warranting a deeper investigation into its role as both a biomarker and potential therapeutic target (3). On this basis, the relationship between carotid atherosclerosis and Lp(a) and other serum lipid parameters in patients with AIS is investigated in the present study to provide evidence for stroke prevention.

## 2 Materials and methods

### 2.1 Study participants

This was a retrospective case-control study. It was pre-registered in Hebei renqiu Kangjixintu Hospital and conducted in accordance with the Declaration of Helsinki and approved by the ethics committee of Hebei renqiu Kangjixintu Hospital. The approval reference number is 20140305-01. A total of 2,018 patients with AIS were selected from the “acute stroke intervention and secondary prevention registration database” constructed using the details of patients with acute stroke admitted to the hospital between January 2014 and January 2016. They were all given statin treatment after admission and were given regular statin treatment thereafter. Some patients also received regular antihypertensive drugs. This treatment adhered to the current standards of medical practice. The inclusion criteria were as follows: patients aged >18 years; patients who were clinically diagnosed with new cerebral infarction, with a delay between symptom onset and hospitalisation of <7 days and with complete carotid artery and transcranial Doppler examinations. The exclusion criterion was as follows: patients with tumours, hyperthyroidism, liver disease or renal insufficiency. Family members of the patients signed informed consent forms.

### 2.2 Methods

In patients with acute stroke who were admitted to the hospital within 7 days after onset, AIS was confirmed by head computed tomography or magnetic resonance imaging. General data of the patients, including sex, age, educational level, past medical history (e.g., hypertension, diabetes, CHD, stroke and dyslipidaemia) and histories of smoking and drinking, were collected after admission.

Physical examination: Blood pressure, heart rate, height, body mass and waist circumference were measured. Blood pressure was measured using an Omron electronic blood pressure meter. The bilateral systolic and diastolic blood pressures were measured, and the highest systolic and diastolic blood pressures were recorded as the final readings.

Laboratory tests: Venous blood samples were collected after 12 h of fasting. Fasting blood glucose (mmol/L), Lp(a) (mg/L), total cholesterol (TC) (mmol/L), triglyceride (TG) (mmol/L), low-density lipoprotein cholesterol (LDL-C) (mmol/L) and high-density lipoprotein cholesterol (HDL-C) (mmol/L) were measured using an Olympus AU40 automatic chemistry analyser. The laboratory tests of blood lipids used kits from Beijing Sanopu.

Carotid artery ultrasound: An IU22 colour Doppler ultrasound system with a 5–10 MHz probe was used. The patients were placed in a supine position with bolsters under both shoulders. The head was turned to the opposite side to fully expose the neck to be examined. A longitudinal examination of the vascular wall, vessel diameter, the carotid artery intima-media thickness (IMT) and the presence of plaque and stenosis in the common carotid artery, carotid bifurcation and 2 cm from the origin of the internal carotid artery were carefully observed along the outer edge of the sternocleidomastoid muscle. The IMT was measured at 1.0 mm proximal to the carotid bifurcation. Unified criteria for ultrasound evaluation of carotid arteries were used in this study. An IMT of ≥1.0 mm was defined as IMT thickening; an IMT of ≥1.5 mm with local protrusion and intima thickening that protruded into the lumen and without lumen stenosis was defined as plaque formation (11); carotid artery stenosis was diagnosed using the criteria published by the Society of Radiologists in an ultrasound consensus conference in 2003 (12).

### 2.3 Related definitions and diagnostic criteria

Smokers: Smokers were defined as patients who continuously smoked or cumulatively smoked for more than 6 months in their lifetime, with at least one cigarette per day.

Diabetes: Patients who were definitively diagnosed with diabetes or took medication for this disease before the onset of stroke.

Hypertension: Patients who were definitively diagnosed with hypertension or took medication for this disease before the onset of stroke.

Lipid metabolism disorder: Patients who were definitively diagnosed with lipid metabolism disorder or took medication for this disease before the onset of stroke. Lipid metabolism disorder was diagnosed in patients with at least one of the following: (1) LDL-C > 3.37 mmol/L (130 mg/dL); (2) TG > 1.7 mmol/L (150 mg/dL); (3) HDL-C < 1.04 mmol/L (40 mg/dL).

Overweight: Body mass index ≥24 kg/m^2^.

### 2.4 Statistical analysis

Data were analysed using the statistical software SPSS 18.0. Enumeration data were presented as *n* (%), and initial univariate analyses were conducted to explore the associations between each independent variable (e.g., age, sex, smoking status, history of diabetes, cholesterol levels and Lp[a] levels) and the presence of carotid atherosclerosis. All the numeric form data were normally distributed according to the normal distribution test. No test for outliers was conducted. The chi-squared (*χ*^2^) test was used for categorical variables, and the independent *t*-test or Mann–Whitney U test was used for continuous variables, depending on the distribution of the data. The trend *χ*^2^ test was used for comparison between groups. Measurement data were presented as mean ± standard deviation, those with skew distribution were presented as M (QR) and the Jonckheere–Terpstra test was used for the trend test. To determine the independent predictors of carotid atherosclerosis, a two-tailed multivariate logistic regression analysis was employed. Variables that were statistically significant in the univariate analyses were included in the regression model. Adjusted odds ratios with 95% confidence intervals and *p*-values were reported. A stepwise backward elimination approach was used to retain variables in the model based on the likelihood ratio test with a significance level of removal set at *p* > 0.05. Differences with a *p*-value of <0.05 were considered statistically significant.

## 3 Results

### 3.1 General characteristics of different severities of carotid atherosclerosis

A total of 1730 patients were included, comprising 1,228 men and 502 women, and the incidence of atherosclerotic plaque and stenosis was 80.2%, with 330 (19.1%) patients without plaque and stenosis, 979 (56.6%) patients with plaque and no stenosis and 421 (23.7%) patients with stenosis. Among the 1,228 male patients, 211 (63.9%) had no arterial plaque and stenosis, 678 (69.3%) had plaque and no stenosis and 339 (80.5%) had stenosis. The prevalence of carotid atherosclerosis in men gradually increased with the severity of atherosclerosis, and the severity of arteriosclerosis increased with age. There were statistically significant differences in the prevalence of risk factors, such as smoking, overweight, history of stroke and history of CHD, as well as the levels of lipid parameters among the three groups of patients. The level of Lp(a) increased gradually with the severity of carotid atherosclerosis (Tables 1, 2).

**Table 1:** Baseline information on risk factors for different types of carotid atherosclerosis.

	No plaque (*n* = 330)	With plaque (*n* = 979)	With stenosis (*n* = 421)	*p*-value
Sex (male)	211 (63.9)	678 (69.3)	339 (80.5)	0.000
Age (years)	56.98 ± 11.39	64.86 ± 10.67	69.26 ± 9.805	0.000
**Stroke risk factors, *n* (%)**				
Smoking, *n* (%)	155(47.0)	557(56.9)	258(61.3)	0.000
Hypertension, *n* (%)	209(63.3)	667(68.1)	284(67.5)	0.276
Diabetes, *n* (%)	48(14.5)	188(19.2)	70(16.6)	0.032
Dyslipidemia, *n* (%)	14(4.2)	46(4.7)	20(4.8)	0.755
History of stroke, *n* (%)	78(23.6)	318(32.5)	179(42.5)	0.000
History of CHD, *n* (%)	28(8.5)	103(10.5)	59(14.0)	0.014
Overweight, *n* (%)	103(31.2)	411(41.0)	201(50.1)	0.000

**Table 2:** Baseline information on metabolic parameters of different types of carotid atherosclerosis.

Metabolic parameters	No plaque (*n* = 330)	With plaque (*n* = 979)	With stenosis (*n* = 421)	*p*-value
Blood glucose (mmol/L)	5.42 (4.88,6.18)	5.55 (4.89,6.69)	5.45 (4.9,6.70)	0.429
TC(mmol/L)	4.72 (4.08,5.43)	4.87 (4.19,5.56)	4.87 (4.19,5.57)	0.047
TG(mmol/L)	1.28 (0.89,1.83)	1.29 (0.91,1.82)	1.19 (0.89，1.63)	0.034
HDL-C(mmol/L)	1.11 (0.93,1.35)	1.07 (0.90,1.32)	1.07 (0.88,1.29)	0.012
LDL-C(mmol/L)	2.33 (1.94,3.10)	2.52 (1.99,3.13)	2.49 (2.01,3.11)	0.131
Lp(a)(mmol/L)	163.95 (76.50,279.50)	186.00 (100.80,320.80)	221.00 (128.50,389.45)	0.000

Values are shown as median in the groups. *p*-values are not corrected for multiple comparisons.

### 3.2 Risk factors for carotid atherosclerosis in patients with acute ischemic stroke

Multivariate logistic regression analysis showed that sex, age, smoking, overweight, history of stroke, history of CHD, history of diabetes, Lp(a) and cholesterol were independent risk factors, and HDL was the protective factor for carotid atherosclerotic stenosis in patients with AIS after adjustments for sex, age, smoking, overweight, history of stroke, history of CHD, history of hypertension, history of diabetes, Lp(a), cholesterol, LDL, HDL and TG. Among the blood lipid parameters, Lp(a) and cholesterol were independent risk factors for carotid atherosclerosis, and the risk increased slightly with the severity of atherosclerosis (Table 3).

**Table 3:** Multivariate logistics analysis of risk factors for carotid atherosclerosis.

	With plaque			With stenosis		
	OR	95%CI	*p*-value	OR	95%CI	*p*-value
Sex	0.704	0.525–0.944	0.019	0.432	0.304–0.615	0.000
Age	1.073	1.059–1.086	0.000	1.121	1.104–1.139	0.000
Smoking	1.777	1.334–2.369	0.000	2.000	1.423–2.81	0.000
History of stroke	1.446	1.081–1.936	0.013	2.022	1.448–2.823	0.000
History of CHD	1.632	1.485–1.885	0.026	2.224	1.852–2.821	0.014
History of diabetes	1.832	1.285–2.612	0.001	1.889	1.233–2.894	0.003
Overweight	1.522	1.132–2.000	0.000	1.694	1.232–2.330	0.001
Lp(a)	1.001	1.000–1.002	0.032	1.002	1.001–1.003	0.000
Cholesterol	1.233	1.086–1.401	0.001	1.37	1.184–1.586	0.000
HDL	0.780	0.441–0.889	0.002	0.517	0.331–0.808	0.004

*p*-values are not corrected for multiple comparisons.

## 4 Discussion

There have been no consistent findings on the effect of Lp(a) and other blood lipid parameters on carotid atherosclerosis in patients with AIS. Liqun et al. (13) found that Lp(a) was correlated and increased with the severity of carotid atherosclerosis. Yubao et al. (14) showed that the levels of blood lipid parameters varied in patients with AIS of different atherosclerotic plaque groups; however, the risk of each parameter for atherosclerotic plaque was not further explored. In the study by Haitao (15), the development of carotid atherosclerosis was correlated with TG and LDL in patients with cerebral infarction, and the levels of TG and LDL were increased in these patients complicated with carotid plaque compared with those in patients without carotid plaque (13). Shaogang et al. (16) investigated the correlation of carotid atherosclerosis with serum uric acid, high-sensitive C-reactive protein and Lp(a) in patients with acute cerebral infarction and found that Lp(a) was correlated with the severity of carotid atherosclerosis, which was consistent with the findings of this study.

The results of the present study showed that the prevalence of sex, smoking, overweight, diabetes, history of stroke and history of CHD among other stroke risk factors gradually increased with the severity of carotid atherosclerosis. The levels of Lp(a), cholesterol and LDL increased and the levels of TG and HDL decreased gradually with the severity of arteriosclerosis (*p*_trend_ < 0.05). Multivariate logistic regression analysis showed that sex, age, smoking, overweight, history of stroke, history of CHD, history of diabetes, Lp(a) and cholesterol were independent risk factors, and HDL was the protective factor for carotid atherosclerotic stenosis in patients with AIS after adjustments for sex, age, smoking, overweight, history of stroke, history of CHD, history of hypertension, history of diabetes, Lp(a), cholesterol, LDL, HDL and TG. Among the blood lipid parameters, only Lp(a) and cholesterol were independent risk factors for carotid atherosclerosis. Therefore, it is necessary to strengthen the management of Lp(a) to control atherosclerosis effectively and prevent stroke. The results of a large-scale population study showed that high Lp(a) was correlated with an increased risk of ischemic stroke (17). A recent retrospective case-control analysis found that Lp(a) was positively correlated with ischemic stroke (18). The results of the present study further confirmed that high Lp(a) was an independent risk factor for carotid atherosclerotic stenosis, and the risk of AIS increased in patients with carotid atherosclerotic stenosis who had a high level of Lp(a).

The level of Lp(a) is relatively stable and not affected by factors such as sex, age, diet, smoking, lipid metabolism drugs and environment. The site and mechanism of Lp(a) metabolism in the body are unknown. Studies have shown that the elevated Lp(a) can be treated with nicotinic acid and coenzyme Q10 (19, 20), and polymorphic loci may serve as a target of gene therapy. Therapeutic effects and evidence-based medical evidence on Lp(a) are not sufficiently reported, and there are no drugs that can be widely used to reduce the level of Lp(a). Therefore, future studies with a large sample size are needed.

The limitations of the present study included the small sample size, study design and data interpretation. First, there may be selection bias in the sample source because the patients in this study were selected from a database established in a single medical institution and may not be representative of the general population. Second, there may be potential confounding factors in the study design, such as failure to take into account all possible risk factors or interventions. In addition, only correlations were analysed in the present study since this was a retrospective study and it was impossible to determine the causal relationship. Finally, the interpretation of the results may be affected by other factors that were not considered, such as lifestyle and genetic factors, which may affect the interpretation of the relationship between Lp(a) and carotid atherosclerosis.

In summary, carotid atherosclerosis is correlated with Lp(a) and cholesterol in patients with AIS, and Lp(a) and cholesterol are independent risk factors for carotid atherosclerosis in these patients. Effective reduction of Lp(a) and cholesterol has become an important measure to control carotid atherosclerosis and stroke. There has been significant progress in the development of specific therapies targeting Lp(a). Notably, antisense oligonucleotides, such as AKCEA-Apo(a)-LRx, and siRNA therapies, such as pelacarsen, are currently under clinical trials (21). Furthermore, lifestyle modification and rational use of statins are effective ways to control stroke. Treatment methods and drugs for Lp(a) will be developed with medical progress to effectively control stroke, and it is expected that clinical results will be obtained quickly without affecting the outcomes.

## Data availability statement

The original contributions presented in the study are included in the article/supplementary material, further inquiries can be directed to the corresponding author.

## Ethics statement

The studies involving humans were approved by this study was conducted in accordance with the Declaration of Helsinki and approved by the ethics committee of Renqiu Kangji Hospital. The studies were conducted in accordance with the local legislation and institutional requirements. Written informed consent for participation was not required from the participants or the participants’ legal guardians/next of kin in accordance with the national legislation and institutional requirements. Written informed consent was obtained from the individual(s) for the publication of any potentially identifiable images or data included in this article.

## Author contributions

YoZ: Writing – review & editing, Writing – original draft, Visualization, Project administration, Methodology, Formal analysis, Data curation. ZW: Writing – review & editing, Writing – original draft, Methodology, Formal analysis. RJ: Writing – review & editing, Writing – original draft, Methodology, Formal analysis. YW: Writing – review & editing, Writing – original draft, Methodology, Formal analysis. YaZ: Writing – review & editing, Writing – original draft, Methodology, Formal analysis. KY: Writing – review & editing, Writing – original draft, Project administration, Methodology, Formal analysis, Conceptualization.

## References

[1] Liu L Chen W Zhou H Duan W Li S Huo X. Chinese Stroke Association guidelines for clinical management of cerebrovascular disorders: executive summary and 2019 update of clinical management of ischaemic cerebrovascular diseases. Stroke Vasc Neurol. (2020) 5:159–76. doi: 10.1136/svn-2020-000378, PMID: 32561535 PMC7337371

[2] Yan X Liu Z Guo ZN Sun Y Jin H Sun X. Positive influence of stroke health manager on risk factors control and medication adherence after ischemic stroke. Front Neurol. (2020) 11:168. doi: 10.3389/fneur.2020.00168, PMID: 32218766 PMC7078325

[3] Di Fusco SA Arca M Scicchitano P Alonzo A Perone F Gulizia MM. Lipoprotein(a): a risk factor for atherosclerosis and an emerging therapeutic target. Heart. (2022) 109:18–25. doi: 10.1136/heartjnl-2021-320708, PMID: 35288443

[4] Chan DC Watts GF Coll B Wasserman SM Marcovina SM Barrett PHR. Lipoprotein(a) particle production as a determinant of plasma lipoprotein(a) concentration across varying apolipoprotein(a) isoform sizes and background cholesterol-lowering therapy. J Am Heart Assoc. (2019) 8:e011781. doi: 10.1161/JAHA.118.011781 30897995 PMC6509712

[5] Kamstrup PR Benn M Tybjaerg-Hansen A Nordestgaard BG. Extreme lipopro-tein(a)levels and risk of myocardial infarction in the general population: the Copenhagen City heart study. Circulation. (2008) 117:176–84. doi: 10.1161/CIRCULATIONAHA.107.715698, PMID: 18086931

[6] Emerging risk factors collaboration, Erqous, Kaptoges. Liporotein(a) concentration and the risk of coronary heart disease, stroke and nonvascular mortality. JAMA. (2009) 302:412–23. doi: 10.1001/jama.2009.1063 19622820 PMC3272390

[7] Nordestgaard BG Chapman MJ Ray K Borén J Andreotti F Watts GF. Lipoprotein(a) as a cardiovascular risk factor: current status. Eur Heart J. (2010) 31:2844–53. doi: 10.1093/eurheartj/ehq386, PMID: 20965889 PMC3295201

[8] Dhamija RK Gaba P Arora S Kaintura A Kumar M Bhattacharjee J. Homocysteine and lipoprotein(a) correlation in ischemic stroke patients. J Neurol Sci. (2009) 281:64–8. doi: 10.1016/j.jns.2009.02.341, PMID: 19285692

[9] Boden-Albala B Kargman DE Lin IF Paik MC Sacco RL Berglund L. Increased stroke risk and lipoprotein(a) in a multiethnic community: the northern Manhattan stroke study. Cerebrovasc Dis. (2010) 30:237–43. doi: 10.1159/000319065, PMID: 20664256 PMC3064933

[10] Zhu L Zheng J Gao B Jin X He Y Zhou L. The correlation between lipoprotein(a) elevations and the risk of recurrent cardiovascular events in CAD patients with different LDL-C levels. BMC Cardiovasc Disord. (2022) 22:171. doi: 10.1186/s12872-022-02618-5, PMID: 35428179 PMC9013030

[11] McLaughlin MM Ma Y Scherzer R Rahalkar S Martin JN Mills C. Association of Viral Persistence and Atherosclerosis in adults with treated HIV infection. JAMA Netw Open. (2020) 3:e2018099. doi: 10.1001/jamanetworkopen.2020.18099, PMID: 33119103 PMC7596582

[12] Grant EG Benson CB Moneta GL Alexandrov AV Baker JD Bluth EI. Carotid artery stenosis:grayscale and Doppler ultrasound diagnosis-society of radiologists in ultrasound consensus conference. Ultrasound Q. (2003) 19:190–8. doi: 10.1097/00013644-200312000-00005 14730262

[13] Liqun C. Study on the relationship between fibrinogen, lipoprotein a and C-reactive protein and carotid artery sclerosis in patients with cerebral infarction. J Clin Exp Med. (2010) 9:810–1.

[14] Yubao F. Correlation analysis of cerebral infarction with blood lipids and carotid atherosclerotic plaque. Clin Med. (2015):58–9.

[15] Haitao L. Relationship between carotid atherosclerotic plaque and levels of plasma-lipid in the patients with cerebral infarction. China Med Pharmacy. (2015) 5:138–9.

[16] Zhang Shaogang W Qiuzong JF. Relationship between carotid atherosclerosis and serum uric acid, high-sensitivity c-reactive protein and lipoprotein(a) in patients with acute cerebral infarction. J Clin Med Practice. (2014) 18:14–6. doi: 10.7619/jcmp.201407004

[17] Langsted A Nordestgaard BG Kamstrup PR. Elevated lipoprotein(a) and risk of ischemic stroke. J Am Coll Cardiol. (2019) 74:54–66. doi: 10.1016/j.jacc.2019.03.524 31272552

[18] Kubota Y Folsom AR Ballantyne CM Tang W. Lipoprotein(a) and abdominal aortic aneurysm risk: the atherosclerosis risk in communities study. Atherosclerosis. (2018) 268:63–7. doi: 10.1016/j.atherosclerosis.2017.10.017, PMID: 29182987 PMC5788200

[19] Stulnig TM Morozzi C Reindl-Schwaighofer R Stefanutti C. Looking at Lp(a) and related cardiovascular risk: from scientific evidence and clinical practice. Curr Atheroscler Rep. (2019) 21:37. doi: 10.1007/s11883-019-0803-9, PMID: 31350625

[20] Momtazi-Borojeni AA Katsiki N Pirro M Banach M Rasadi KA Sahebkar A. Dietary natural products as emerging lipoprotein(a)-lowering agents. J Cell Physiol. (2019) 234:12581–94. doi: 10.1002/jcp.28134, PMID: 30637725 PMC13484089

[21] Tsimikas S Karwatowska-Prokopczuk E Gouni-Berthold I Tardif JC Baum SJ Steinhagen-Thiessen E. Lipoprotein(a) reduction in persons with cardiovascular disease. N Engl J Med. (2020) 382:244–55. doi: 10.1056/NEJMoa1905239 31893580

